# Inhibitory effects of pu-erh tea on alpha glucosidase and alpha amylase: a systemic review

**DOI:** 10.1038/s41387-019-0092-y

**Published:** 2019-08-27

**Authors:** Chiung-Ying Yang, Yea-Yin Yen, Kuang-Chen Hung, Shang-Wei Hsu, Shou-Jen Lan, Hsin-Cheng Lin

**Affiliations:** 1 Department of Healthcare Administration, Asia University, Taiwan, Taiwan; 2 Department of Oral Hygiene, College of Dental Medicine, Kaohsiung Medical University, Taichung, Taiwan; 3 Central Taiwan University of Science and Technology, Taichung, Taiwan; 4 Taichung Armed Forces General Hospital, Taichung, Taiwan; 5 National Defense Medical Center, Taipei, Taiwan; 6 Department of Medical Research, China Medical University Hospital, China Medical University, Taichung, Taiwan

**Keywords:** Preclinical research, Diseases

## Abstract

**Objective:**

Pu-erh tea was presumed to have anti-hyperglycemic effects via inhibition on alpha-amylase and alpha-glucosidase. However, no integerated literatures were published to substantiate such presumption.

**Methods:**

Current study adopted systemic review method to validate inhibitory effects on alpha amylase and alpha-glucosidase. Five English databases (PubMed, EBSCO, SCOPUS, Cochrane Library, Web of Science) and three Chinese ones (Airti Library, CNKI Library, and Google Scholar) were searched up to 22 March 2018 for eligible literatures, using keywords of Pu-erh, Pu’er, alpha-amylase or alpha-glucosidase.

**Results:**

Six studies exploring inhibitory effects on alpha-glucosidase and seven on alpha-amylase were included for systemic review. Though results showed pu-erh tea has significant inhibitory effects on alpha-amylase and alpha-glucosidase, high heterogeneity was detected among studies included.

**Conclusions:**

High heterogeneity may be due to complex alterations of chemicals under different degrees of fermentation. More future studies are required to further identify principal bioactive component(s) at work.

## Introduction

Tea drinking has been part of Chinese culture for centuries. Tea was widely regarded to have many health benefits. Green tea, black tea, or oolong teas were all good for rats on high fructose diet. Pu-erh tea is made from camellia sinensis plant’s leaves and stems, also the same principal ingredient for green, oolong, and black teas. Different teas were processed and manufactured using distinct preparation procedures, where green tea is not fermented, oolong partially fermented, black tea fully fermented, and pu-erh post-fermented. Pu-erh tea has long been espoused as a health beverage in oriental societies including China and Taiwan. China’s Compendium of Materia Medica contains passages of: “Pu-erh tea taste bitter and promote fat metabolism. Moreover the Pu-erh tea paste looks like black paint, it can sober up the drunken people.”

Pu-erh tea is primarily grown in Yunnan Province of China. Pu-erh tea extracts were found to significantly improve plasma concentrations of glucose, insulin, triglycerides, and free fatty acids^1–3^. Recent studies have further confirmed that catechins, caffeine, polyphenols, amino acids, and polysaccharides in pu-erh tea extracts also have beneficial effects on maintaining in vivo glucose homeostasis in type 2 diabetes mice^4^. Pu-erh tea’s distinctive effects could potentially be attributed to a series of complex changes in chemicals during post-fermentation stage. These complex chemical changes in turn were found to lead to pu-erh’s great health benefits, such as lower atherosclerotic risk^5^, weight reduction^6^, and anti-hyperglycaemic effect, especially^7^.

**Fig. 1:**
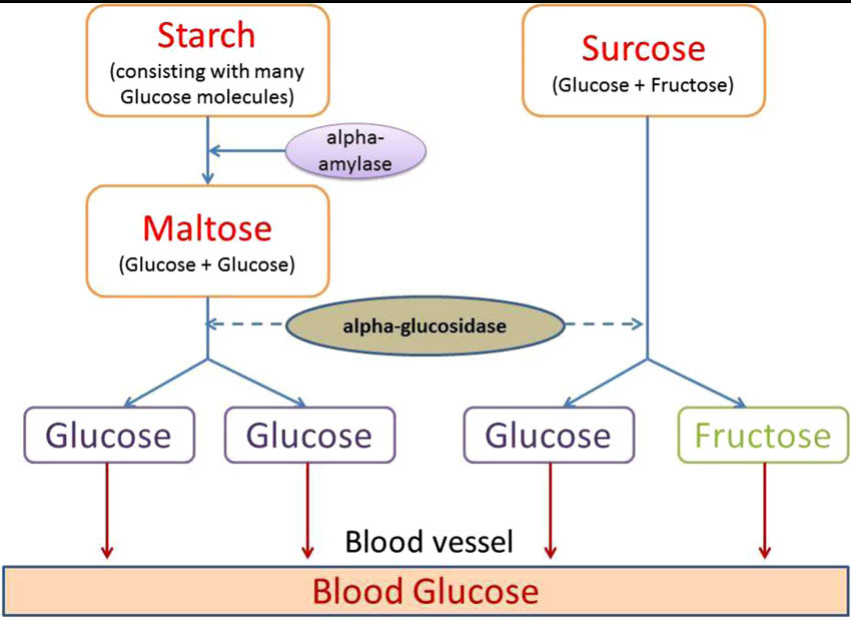
Hydrolysis of starch to glucose as catalyzed by alpha-amylase and alpha-glucosidase

Starch is the most important source of carbohydrates for human and several different animal species while α-amylase and α-glucosidase are two primary enzymes involved in carbohydrate digestion. Salivary and pancreatic α-amylases cleave the α-(1 → 4)-D-glycosidic bonds of starch at random sites, forming smaller oligosaccharides or disaccharides. Alfa glucosidase is located in brush borders of small intestines, where this digestive enzyme hydrolyzes terminal non-reducing α-1 → 4 linkage of oligosaccharides or disaccharides and releases glucose molecules (Fig. 1).

Alpha glucosidase plays an important role in regulating postprandial blood glucose levels in human body^8^ and its inhibitors, capable of suppressing postprandial hyperglycaemia, are usually used to prevent or treat type II diabetes^9^. Alpha amylase is basically a calcium metalloenzyme, acting as a catalyst in reactions involving alpha-1,4 glycosidic linkages hydrolyses of starch, amylopectin, amylose, glycogen, and numerous maltodextrins, and is therefore responsible for starch digestion. In order to make use of starch, the human digestive system, with the help of the enzyme amylases, break down the polymer to smaller assimilable sugars, which is eventually converted to the individual basic glucose units. Under some circumstances, such as excess activity of amylase enzymes, insulin deficiency, or resistance to insulin, blood glucose level may rise and in turn result in hyperglycaemia.

Carbohydrates from pu-erh tea polysaccharides (PTPS) can inhibit both alpha glucosidase and alpha amylase^10^. To offer alternative or even better solutions for diabetes patients to control their blood sugar level, this study aims to explore pu-erh tea’s effects on diabetes mellitus. Adopting systemic literature review method and analyzing all eligible research articles included for analysis, this study investigated pu-erh tea’s inhibitory effects on alpha-glucosidase and alpha-amylase in lowering blood glucose and made comprehensive conclusions regarding pu-erh tea’s effectiveness on regulating blood sugar level.

## Method

Systemic literature review method was used to determine inhibitory effects of pu-erh tea on alpha-glucosidase and alpha-amylase.

### Information sources and searches

Eight bibliographic databases were searched: (1) PubMed, (2) EBSCO, (3) SCOPUS, (4) Cochrane Library, (5) Web of science, (6) Airiti Library, (7) China National Knowledge Infrastructure (CNKI), and (8) Google Scholar. Among the eight databases, Airiti Library and China National Knowledge Infrastructure (CNKI) were searched for literatures in Chinese. Furthermore, Google Scholar was also searched for eligible studies either in English or Chinese. The Search terms are listed in Table 1.

**Table 1:** Search terms

Search Strings	AND	Search strings
Pu-erh		Alpha glucosidase
OR		OR
Pu’er		Alpha amylase

### Study selection

A total of 71 articles were initially retrieved from the eight databased searched (five English databases, two Chinese databases, and Google Scholar searching for articles either in English or Chinese).

Out of the 71 articles retrieved, 45 of them were retained after removing 16 duplicate or review articles. Current study authors further excluded 32 articles deemed unsuitable for systemic review by examining their respective title and abstract.

The remaining 13 articles were further reduced to 12, after excluding 0 studies determined irrelevant to the purpose of the current study and one non full-text articles.

For the remaining 12 full-text articles selected after abovementioned screenings, all of them were unanimously determined by all research authors to pass PICO (population, interventions, compare, and outcome) criteria and were in turn included for full review.

It is noteworthy that 1 study Du, Peng^4^ included for full review was determined to relate to inhibition effects on both alpha amylase and alpha glucosidase, consequently making the final full-review articles count to 13 from 12.

Also noteworthy is that a trial was considered eligible and in turn included for full review if it addressed any pu-erh tea intervention effects, whether in vivo or in vitro. The outcome column in Table 2 highlighted inhibition rate of alpha-amylase or alpha-glucosidase of individual studies included for full review. Below excluding criteria were employed to remove ineligible studies: review article, duplicate article, non-full-text article, study without data on blood glucose change, and non-animal trial.

**Table 2:** System review results of Pu-erh tea inhibition effects on alpha glucosidase

Study	Experiment subjects	outcome
Li et al.^11^	Alpha glucosidase inhibition assay	1. (−)-epigallocatechingallate (EGCG) and (−)-epicatechingallate (ECG), as potent alpha glucosidase inhibitors, were found in pu-erh tea. 2. The IC_50_ values of EGCG and ECG on alpha-glucosidase were 175.1 and 246.9 μM, respectively, and both were lower than that of acarbose (IC_50 _= 3553.0 μM), a commercial alpha glucosidase inhibitor. 3. Different concentrations have different Inhibition rates (1.0 17.1 + −1.3).
Yang et al.^12^	Alpha glucosidase inhibition assay	In the concentration 2.5 mg/ml, the inhibition rate of acarbose: 60.16%, ethyl acetate fraction: 82.52%, n-butanol fraction: 65.84%, water fraction: 58.22%, water extract: 52.29%.
Du et al.^4^	Alpha glucosidase inhibition assays for the WEPT were conducted using rat small intestinal sucrose and maltase in vitro	WEPT showed inhibitory effects on rat intestinal sucrose, maltase, and porcine pancreatic amylase, as shown in Table 3, but are less potent compared to acarbose (IC_50 _= 4.35 ± 0.59, 6.63 ± 0.70, and 64.19 ± 6.77 μmol/L for sucrose, maltase, and amylase, respectively).
Huang et al.^13^	Alpha glucosidase inhibition assay	1. EP exhibited the strongest inhibitory potential, which increased from 25.96% to 87.2% when the concentration increased from 4 μg/ml to 125 μg/ml. 2. EF, the inhibitory effect increased from 20.43% to 84.77% at the same range of concentrations. 3. Meanwhile, AE, R and SF also showed a great inhibition on a-glycosidase with an inhibitory effect of 78.85%, 69.94% and 69.47% at a concentration of125ug/ml, which were higher than that of acarbose at the same concentration (40.84%).
Deng et al.^10^	Enzyme assay of α-glucosidase activity	1. The IC50 of acarbose and the IC50s of TPSs from Feng Huang Dan Cong, Da Hong Pao and pu-erh tea (1-year old, 3-year old and 5-year old) on alpha glucosidase activity were 207.195, 5.653, 2.286, 2.192, 0.583, and 0.438 μg/ml. 2. PTPS had more significant effects on α-glucosidase than acarbose did.
Wang et al.^14^	Alpha glucosidase inhibition assay	1. The results showed that EtOAc fraction had a moderate inhibitory activity with IC_50_ values of 14.4 μg/mL against sucrose and 11.4 μg/mL against maltase, respectively. 2. The inhibitory effects of compounds 1–17 were also measured using the same methods. (–)-Epigallo-catechin-3-O-gallate (15) showed a moderate activity with IC_50_ values of 32.5 μmol/L against sucrose and 1.3 μmol/L against maltase, respectively.

## Results

### Search results

In the first stage of the literature search, a total of 71 studies were identified. Among the 71 potentially eligible articles initially searched, 21 articles were published in English (2 from PubMed, 2 from EBSCO, 5 from SCOPUS, 0 from Cochrane, 1 from Web of Science, and 11 from Google Scholar) and 50 studied were published in Chinese. (7 from Airti Library, 9 from China National Knowledge Infrastructure (CNKI), and 34 from Google Scholar.

The second stage was employed to further examine searched articles and determine their respective eligibility, per inclusion and exclusion criteria jointly agreed by all research authors, with duplicate or ineligible articles removed from being included in final review accordingly.

**Fig. 2:**
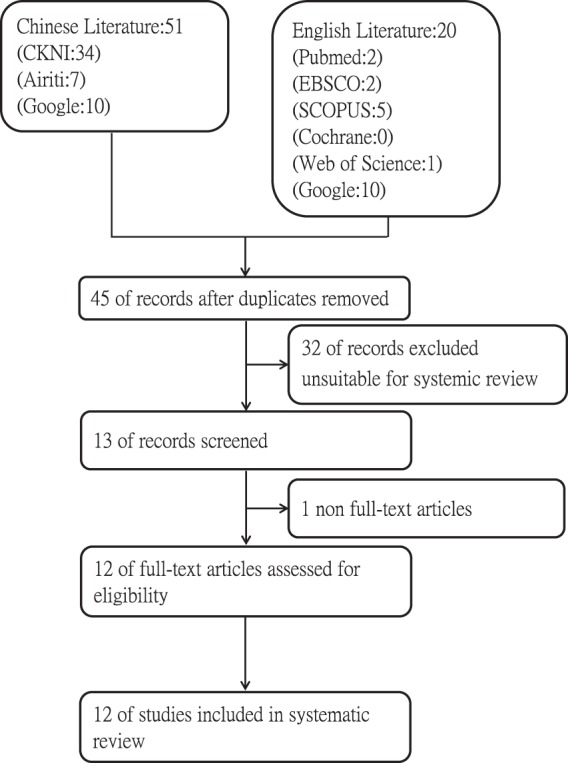
Flow chart describing literature extraction process

After the aforementioned search strategy and two rounds of eligibility screening process, a total of 12 articles were selected and included in the current study’s systemic review: six studies exploring pu-erh tea’s inhibitory effects on alpha-glucosidase and another seven studies regarding its inhibitory effects on α-amylase (Fig. 2).

### Inhibitory effects on α-glucosidase

Li et al.^11^ examined inhibitory effects on alfa glucosidase of EGCG and ECG, two compounds found in pu-erh tea and their activities expressed in dose-dependent manner as opposed to those of acarbose, a commercial alpha-glucosidase inhibitor anti-diabetic drug used to treat type 2 diabetes. IC_50_ values of EGCG and ECG were determined to be 175.1 and 246.9 μM respectively, much lower than that of acarbose (3553.0 μM). Kinetic studies further revealed that both EGCG and ECG inhibit alpha-glucosidase in a non-competitive manner.

Yang et al.^12^ researched water extract of pu-erh tea (WEPT) and its fractions extracted by chloroform, ethyl acetate, n-butanol, and water. Research results showed that pu-erh’s fractions had different inhibitory effects on alpha-glucosidase activity when their concentration was 2.5 mg/ml. Positive controls of their study: acarbose, ethyl acetate fraction, n-butanol fraction, water fraction, and water extract revealed a noticeable difference in inhibitory activity, with inhibition rates of 60.16%, 82.52%, 65.84%, 58.22%, and 52.29% respectively. Varying effects of water extract and its different extraction fractions from pu-erh tea on alpha glucosidase activity were found to be dictated by difference in chemical composition.

Du et al.^4^ used high-performance liquid chromatography and colorimetric methods to analyze tea catechins, caffeine, polyphenols, amino acids, and polysaccharides of WEPT. In this study, α-glucosidase inhibition assays for WEPT were conducted, using rat small intestinal sucrase and maltase in vitro. In their study, WEPT was found to have inhibitory effects on rat intestinal sucrase, maltase, and porcine pancreatic amylase (as shown in Table 3), but WEPT’s inhibitory effects were found to be less potent than those of acarbose. (IC_50_ = 4.35 ± 0.59, 6.63 ± 0.70, and 64.19 ± 6.77 μmol/L for sucrase, maltase, and amylase respectively) The results showed that WEPT dose-dependently and significantly increased glucose uptake by HepG2 cells and inhibited rat intestinal sucrase, maltase, and porcine pancreatic amylase activity.

**Table 3:** System review results of pu-erh’s inhibition effects on alpha amylase

Study	Experiment subjects	Outcome
Zhang et al.^15^	Alpha amylase inhibition assay	Alfa-amylase inhibition of raw materials increased with the extension of fermentation time, the inhibition rate dropped, and reached the lowest in mid stage fermentation, while in late fermentation, inhibition increased and αamylase inhibition of product tea was stronger.
Zhang et al.^16^	Alpha amylase inhibition assay	The results showed that the best inhibition rate is 32.68% in water temperature of 90 °C, pu-erh tea/water ratio of 1:20, and extraction time lasting 1 h.
Zhang et al.^17^	Alpha amylase inhibition assay	The result showed that Gallic acid had a strong inhibition on alpha amylase, with inhibition rate of 63.76%.
Du et al.^4^	Alpha glucosidase inhibition assays for the WEPT were conducted using rat small intestinal sucrose and maltase in vitro	WEPT showed inhibitory effects on rat intestinal sucrose, maltase, and porcine pancreatic amylase, as shown in Table 3, but are less potent compared to acarbose (IC_50 _= 4.35 ± 0.59, 6.63 ± 0.70, and 64.19 ± 6.77 μmol/L for sucrose, maltase, and amylase, respectively).
Zhou et al^18^	Alpha amylase inhibition assay	1. Prolonged storage years will increase inhibition effect on alpha amylase activity. 2. The inhibition rate ranges from 10 to 22%. The regression model: *y* (alphaamylase inhibition rate) = −31.43 + 0.748x; X: theabrownin, TB; *R*^2^ = 97.4%.
Liu and Huang^19^	Alpha amylase inhibition assay	1. Pu-erh has inhibition effects on alpha amylase. 2. Regression equation: *y* = 0.0008x3 + 0.0075x2–0.0106x+ 0.1236, X: extracted tea concentration; *R*^2^ = 0. 998.
Wu et al.^20^	Alpha amylase inhibition assay	Optimal temperature of alpha amylase inhibitor acting is 70 °C, and suppression ratio of alpha Amylase inhibitor in these teas is Ilex >aged pu-erh tea >extra-strong tea >dragon well tea >fresh pu-erh tea.

Huang et al.^13^ established a procedure to obtain pu-erh’s aqueous extract and its fraction enriched with active constituents. The authors also used this procedure to evaluate bioactivities of pu-erh’s aqueous extract and its fractions, including antioxidant activity and inhibitory potential against alpha-glycosidase in vitro as well as effect on postprandial hyperglycemia in alloxan-induced diabetic mice. Their study also found that tea polyphenols, tea polysaccharides, caffeine, protein, amino acids were accumulated in several fractions after solvent extraction despite not being separated completely. Furthermore, 95% ethanol precipitate and ethyl acetate fraction possessed remarkable antioxidant activities and potent inhibitory effects on alpha glycosidase in vitro. The authors also observed that all extracts showed a concentration-dependent inhibition on a-glycosidase. Study results further showed that EP exhibited the strongest inhibitory potential, increasing from 25.96 to 87.2% with concentration level increasing from 4 μg/ml to 125 μg/ml. EF was found to rank second in terms of inhibitory effect which increased from 20.43 to 84.77% at the same range of concentrations. The authors further found that AE, R, and SF also showed a great inhibition on a-glycosidase with an inhibitory effect of 78.85, 69.94, and 64.97% at a concentration of 125 μg/ml, much higher than that of acarbose at the same concentration (40.84%). Results derived from assay performed also showed that CF exhibited the weakest inhibitory potential against a-glycosidase, with highest inhibition of 36.94% at a concentration of 125 μg/ml.

Deng et al.^10^ detected a group of carbohydrates found in PTPS that can inhibit alpha-glucosidase. When comparing inhibition effects of PTPS and acarbose on alpha glucosidase, they found that PTPS from pu-erh tea (1-year old, 3-year old, and 5-year old) significantly inhibited alpha glucosidase activity but did not affect alpha amylase activity. In comparing inhibitory effects on alpha glucosidase activity of acarbose and PTPS found in different types of teas, their study have identified their respective IC_50_ values to be: (1) 207.195 μg/ml for acarbose, (2) 5.653 μg/ml for Feng Huang Dan Cong (special types of oolong from Feng Huang Shan or “Phoenix Mountains” in Guandong province, China.), (3) 2.286 μg/ml for Da Hong Pao (a heavily oxidized, dark oolong tea), (4) 2.192 μg/ml for 1-year old pu-erh tea, (5) 0.583 μg/ml for 3-year old pu-erh tea, and (6) 0.438 μg/ml for 5-year old pu-erh tea. Results from their study have clearly indicated that PTPS had more significant effects on alpha glucosidase than did acarbose.

Wang et al.^14^ found that WEPT showed potential in vitro hypoglycemic effects. The results showed that EtOAc fraction had moderate inhibitory activity with IC_50_ values of 14.4 μg/mL against sucrose and 11.4 μg/mL against maltase (Acarbose IC_50_ values of 0.262 μg/mL against sucrose and 0.084 μg/mL against maltase).

### Inhibitory effect on α-amylase

Zhang et al.^15^ established that alpha amylase inhibition of raw pu-erh tea increased with the extension of fermentation time. The inhibition rate dropped, and reached the lowest in mid stage fermentation, while in late fermentation, the inhibition increased and α-amylase inhibition of product tea was stronger, with inhibition rates ranging from 45.61 to 12.44%.

Zhang et al.^16^ examined the relationship between water temperature, pu-erh tea/water ratio, extraction time, and amylase inhibition rate. The results showed that the best inhibition rate is 32.68%, with water temperature of 90°C, pu-erh tea/water ratio being 1:20, and extraction time lasting 1 h respectively.

Zhang et al.^17^ explored chemical constituents of Pu-erh tea and its inhibition effect on α-amylase. Uracil and gallic acids were isolated and identified respectively from pu-erh tea. The result showed that gallic acid had a strong inhibition on alpha amylase, with inhibition rate of 63.76%.

Zhou et al.^18^ looked into quality change of pu-erh tea, using a spectrophotometer to evaluate inhibition effects on a-amylase of pu-erh teas with different storage years. Results indicated that with storage year prolonging, its inhibition effect on alpha amylase activity also increased, with inhibition rate ranging from 10 to 22%. The regression model: *y* (alpha-amylase inhibition rate) = −31.43 + 0.748x (x: theabrownin or TB); *R*^2^ is 97.4%.

Liu et al.^19^ concluded that pu-erh has an inhibition effect on alpha amylase, with its regression equation shown as: *y* = 0.0008x^3 + ^0.0075x^2^ − 0.010x+ 0.1236 (X: extracted tea concentration); *R*^2 ^= 0. 998.

Wu et al.^20^ based their study on alpha-amylase inhibitor in several teas. Their study found below results: (1) Optimal temperature of alpha amylase inhibitor acting is 70 °C, (2) Suppression ratio of alpha Amylase inhibitor in these teas is Ilex > aged pu-erh tea > extra-strong tea > dragon well tea > fresh pu-erh tea.

Du et al.^4^ used high-performance liquid chromatography and colorimetric methods to analyze tea catechins, caffeine, polyphenols, amino acids, and polysaccharides of WEPT. Results of their research showed that WEPT dose-dependently and significantly inhibited pancreatic amylase activities in both mice and porcine.

## Discussion

### Inhibitory effects on α-glucosidase

Lin et al.^7^ used systemic review and meta-analysis in their study and found that Pu-erh tea can help regulate and maintain adequate level of blood sugar. However, the specific bioactive ingredient(s) or mechanism for lowering hyperglycaemia was still unknown for certainty. According to the authors’ research findings, pu-erh tea’s anti-hyperglycaemia mechanism was associated with the inhibition either on α-glucosidase or α-amylase.

But Pu-erh tea’s inhibitory effects on α-glucosidase as compared to those of Acarbose remained uncertain. Li et al.^11^ and Deng et al.^10^ indicated that pu-erh teas were potent alpha glucosidase inhibitors, with their IC_50_ values much lower than those of acarbose. However, Wang et al.^14^ found that WEPT had only moderate inhibitory activity with IC50 values of 14.4 μg/mL against sucrose and 11.4 μg/mL against maltase (Acarbose IC50 values of 0.262 μg/mL against sucrose and 0.084 μg/mL against maltase).

### Inhibitory effects on α-amylase

Zhang et al.^17^ found that gallic acid had a strong inhibition effect on alpha amylase, with inhibition rate of 63.76%. Findings derived from the authors’ review showed that the inhibition effect was primarily associated with fermentation time, water temperature, pu-erh tea/water ratio, among others.

## Conclusion

With its rapidly increasing prevalence, chronic disease course, and gravely debilitating clinical complications, Diabetes mellitus has become one of the primary threats to human health. As such, postprandial hyperglycemia control has naturally become an urgent issue in prevention, treatment, and control of diabetes.

Alpha-glucosidases are glycoside hydrolases found on luminal surface of enterocytes containing maltase/glucoamylase and sucrase/isomaltase activities. Alpha-glucosidase inhibitors also favorably affected several cardiovascular activities. The risk factors, such as obesity, hypertension, and high glycaemic variability was at little to no risk for hypoglycemia^21^.

More and more studies have been conducted to examine inhibition effects on alpha glucosidase and alpha amylase of pu-erh tea, aimed at exploring physiological and functional results for the prevention and treatment of type 2 diabetes. It is noteworthy that high heterogeneity was detected among these studies. Other studies also indicated that health-promoting benefits of tea drinking could be the summation of effects by all individual ingredients in pu-erh, especially polyphenols. One possible reason is that complex alterations may occur in chemicals under different degrees of fermentation, resulting in an obscure understanding of chemical compositions in pu-erh teas, unlike the much studied and more thoroughly understood green (unfermented) teas. As such, more future studies are required to further identify principal bioactive component(s) at work and their actual mechanisms.
